# Supplementary material for: Identification of gene regulatory networks affected across drug-resistant epilepsies
Source: Nat Commun. 2024 Mar 11;15:2180. doi: 10.1038/s41467-024-46592-2 (PMC10928184; doi:10.1038/s41467-024-46592-2)
Supplement: Supplementary file 1 — Supplementary Information [file 41467_2024_46592_MOESM1_ESM.pdf]

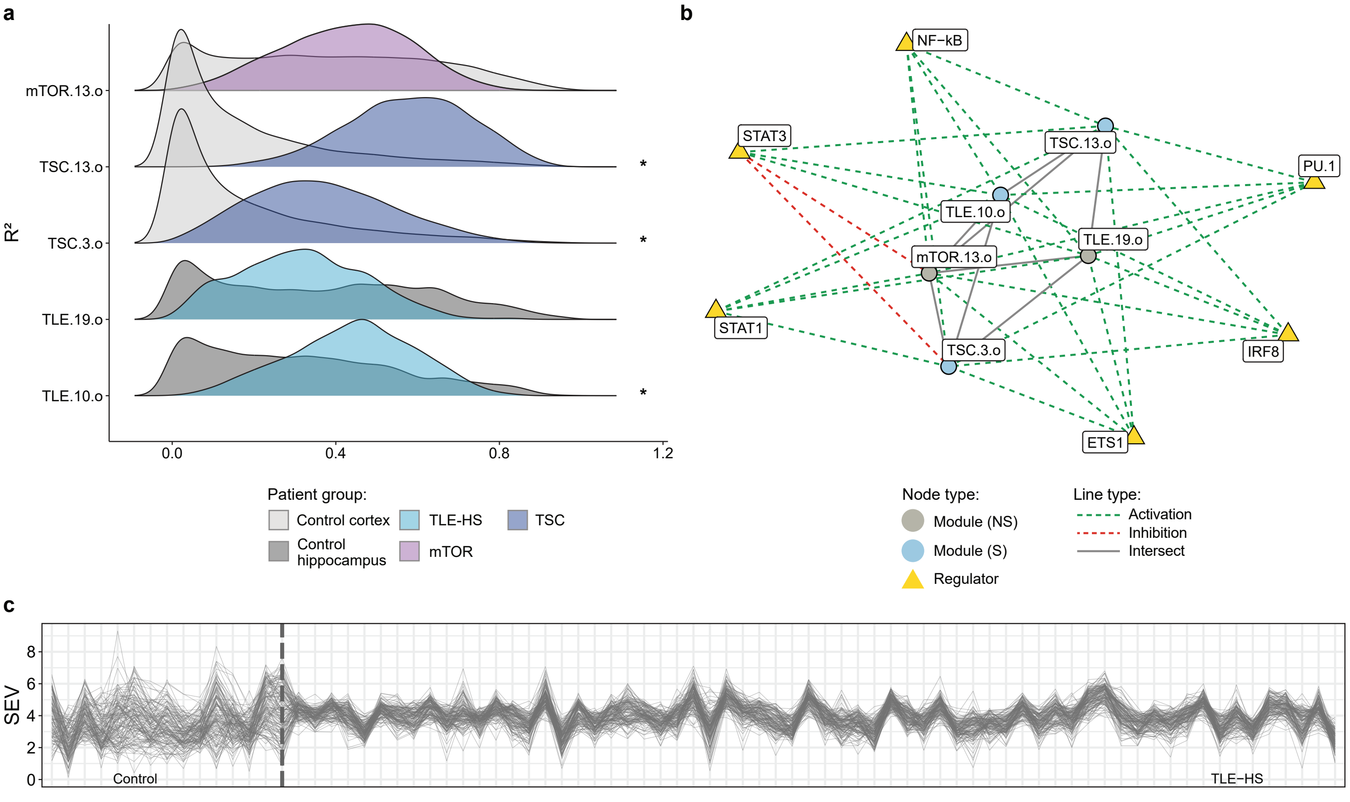
a
mTOR.13.o
TSC.13.o
TSC.3.o
TLE.19.o
TLE.10.o
R²
0.0
0.4
0.8
1.2
*
*
*
Patient group:
Control cortex
TLE-HS
TSC
Control hippocampus
mTOR
b
NF−kB
STAT3
TSC.13.o
PU.1
TLE.10.o
TLE.19.o
mTOR.13.o
STAT1
TSC.3.o
IRF8
ETS1
Node type:
Module (NS)
Module (S)
Regulator
Line type:
Activation
Inhibition
Intersect
c
SEV
0
2
4
6
8
Control
TLE−HS

Supplementary figure legends

Supplementary Fig. 1 | Differential coexpression and upstream transcriptional regulators of the regulomes related to immune response and neuroinflammation. **a,** The ridgeplots show the distribution of gene modules coexpression (R^2) for epilepsy and control patient cohorts within the immune response and neuroinflammation regulomes. **b,** Immune response and neuroinflammation network with indication of differential coexpression of the relevant gene modules. For the immune response regulome, multiple regulators PU.1, STAT1, STAT3, ETS1, IRF8, and NF-kB were predicted as common transcriptional regulators showing activation effect in these gene modules. STAT3 was identified to have a potential inhibitory role for gene modules TSC.3.o and mTOR.13.o. **c,** The line plot shows the standardized expression values for each gene of module TLE.20.o (y-axis) plotted across control samples and TLE-HS samples (x-axis). mTOR, mechanistic target of rapamycin; NS, not significant (differential coexpression P-value > 0.05); S, significant (differential coexpression P-value ≤ 0.05); TLE-HS, temporal lobe epilepsy with hippocampal sclerosis; TSC, tuberous sclerosis complex.

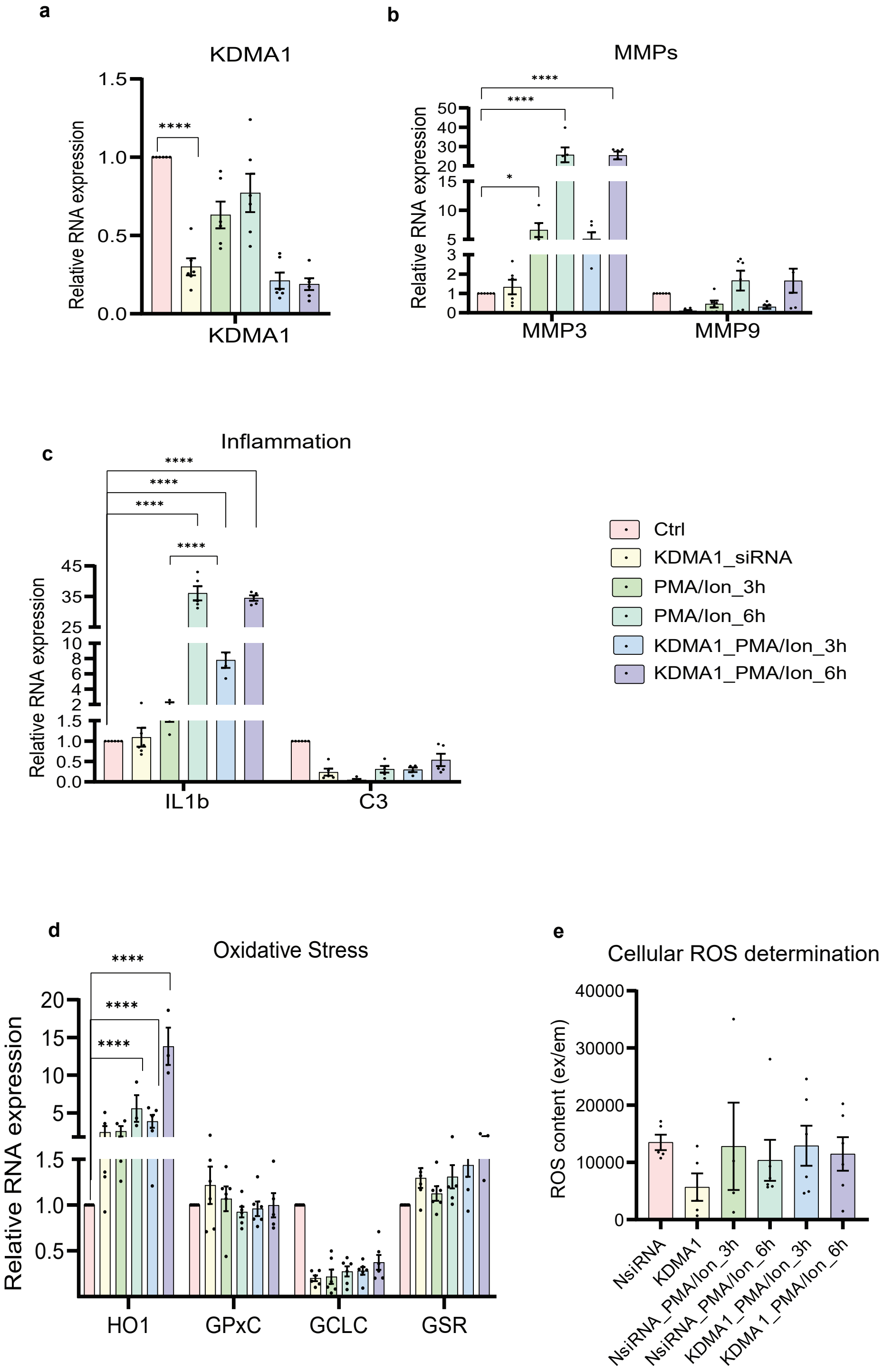
a
KDMA1
Relative RNA expression
KDMA1
****
b
MMPs
Relative RNA expression
MMP3
MMP9
****
*
c
Inflammation
Relative RNA expression
IL1b
C3
Ctrl
KDMA1_siRNA
PMA/Ion_3h
PMA/Ion_6h
KDMA1_PMA/Ion_3h
KDMA1_PMA/Ion_6h
d
Oxidative Stress
Relative RNA expression
HO1
GPxC
GCLC
GSR
e
Cellular ROS determination
ROS content (ex/em)
NsiRNA
KDMA1
NsiRNA_PMA/Ion_3h
NsiRNA_PMA/Ion_6h
KDMA1_PMA/Ion_3h
KDMA 1_PMA/Ion_6h

Supplementary Fig. 2 | Assessment of functional role of KDM1A in PMA/Ionomycin stimulated fetal astrocytes at 3h and 6h (n=3 biological replicates, n=2 technical replicates). a, KDM1A is downregulated (adj. p-value < 0.0001) after KDM1A siRNA inhibition in both control and PMA/Ionomycin stimulated cells (3h/6h). One-Way ANOVA, non parametric testing: Krustal-Wallis statistical test, Dunn's multiple comparisons test. **b,** Stimulation of PMA/Ionomycin confirmed upregulation of *MMP3* (adj. p-value = 0.0135; adj. p-value < 0.0001; adj. p-value < 0.0001) and *MMP9*. **c,** KDM1A siRNA inhibition induced a significant upregulation of *IL1b* (adj. p-value < 0.0001) after 3h of PMA/Ionomycin stimulation but no significant change in *C3* expression. **d,** KDM1A downregulation after 3h of PMA/Ionomycin stimulation did not induce changes in the expression of ROS markers compared to KDM1A downregulation alone (adj. p-value < 0.0001). **e,** KDM1A downregulation after 3h of PMA/Ionomycin stimulation did not provide significant changes in cellular ROS production in astrocytes. Data are expressed as mean ± SEM. Two-Way ANOVA, Tukey's multiple comparisons test. Adjusted p-value: * p.adj ≤ 0.05; ** p.adj ≤ 0.01; **** p.adj ≤ 0.0001.

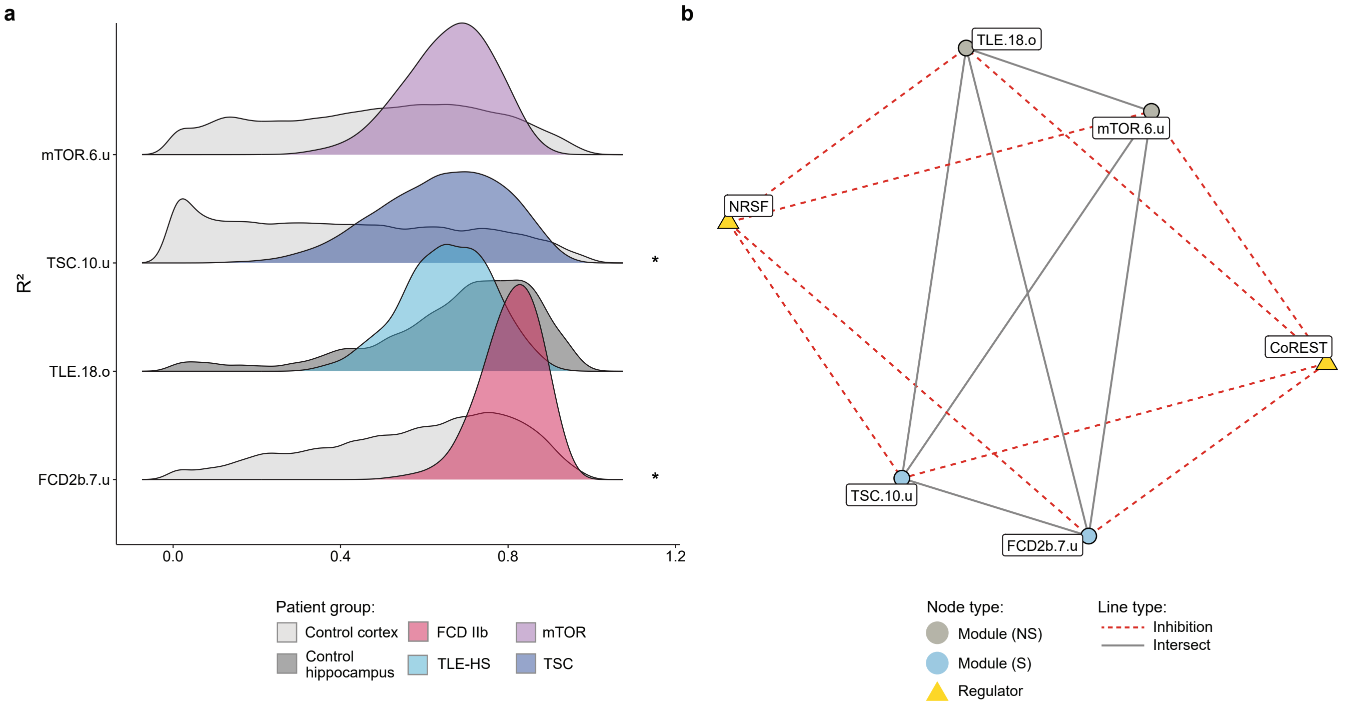

a
R²
mTOR.6.u
TSC.10.u
TLE.18.o
FCD2b.7.u
*
*
0.0
0.4
0.8
1.2
Patient group:
Control cortex
Control hippocampus
FCD IIb
TLE-HS
mTOR
TSC
b
TLE.18.o
mTOR.6.u
NRSF
CoREST
TSC.10.u
FCD2b.7.u
Node type:
Module (NS)
Module (S)
Regulator
Line type:
Inhibition
Intersect

Supplementary Fig. 3 | Differential coexpression and upstream transcriptional regulators of the regulomes related to neuronal function. a, The ridgeplots show the distribution of gene modules coexpression (R^2) for epilepsy and control patient cohorts within the neuronal function regulome. **b,** NRSF and CoREST were predicted for all modules. All transcriptional regulations were predicted to have an inhibitory role on the genes under their regulation. FDC IIb, focal cortical dysplasia type IIb; mTOR, mechanistic target of rapamycin; NS, not significant (differential coexpression P-value > 0.05); S, significant (differential coexpression P-value ≤ 0.05); TLE-HS, temporal lobe epilepsy with hippocampal sclerosis; TSC, tuberous sclerosis complex.

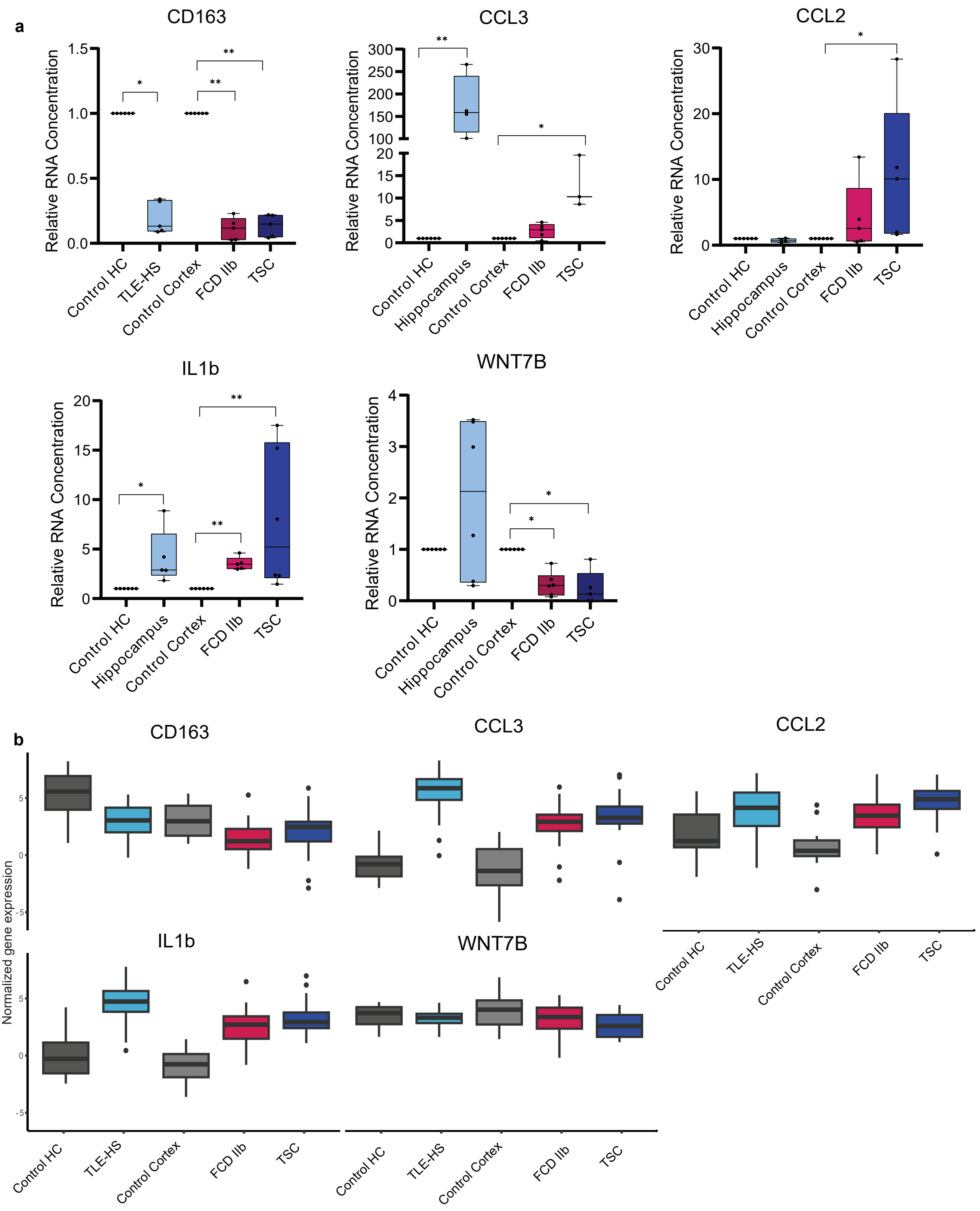

a
CD163
CCL3
CCL2
IL1b
WNT7B
Relative RNA Concentration
Control HC
TLE-HS
Hippocampus
Control Cortex
FCD IIb
TSC
b
CD163
CCL3
CCL2
IL1b
WNT7B
Normalized gene expression
Control HC
TLE-HS
Control Cortex
FCD IIb
TSC

Supplementary Fig. 4 | Expression profiles of five selected genes in all independent cohorts with qPCR (n = 6 per cohort, n=2 technical replicates) and RNAseq. a, qPCR validation of genes *CD163* (Hippocampus: adj. p-value = 0.0287; FCD IIb: adj. p-value = 0.0035; TSC: adj. p-value =0.0085), *CCL3* (Hippocampus: adj. p-value = 0.0013, TSC: adj. p-value = 0.0419); *CCL2* (TSC: adj. p-value =0.0397), *IL1B* (Hippocampus: adj. p-value = 0.0185; FCD IIb: adj. p-value = 0.0063; TSC: adj. p-value =0.0057) and *WNT7B* (FCD IIb: adj. p-value = 0.0376 ; TSC: adj. p-value =0.0137). **b,** Normalized expression based on RNAseq for *CD163, CCL3, CCL2, IL1B, WNT7B.* Data are expressed as mean ± SEM. One-Way ANOVA, non parametric testing: Krustal-Wallis statistical test, Dunn's multiple comparisons test. Adjusted p-value: * p.adj ≤ 0.05; ** p.adj ≤ 0.01; **** p.adj ≤ 0.0001.
