# Supplementary material for: Identification of gene regulatory networks affected across drug-resistant epilepsies
Source: Nat Commun. 2024 Mar 11;15:2180. doi: 10.1038/s41467-024-46592-2 (PMC10928184; doi:10.1038/s41467-024-46592-2)
Supplement: Supplementary file 3 — Description of Additional Supplementary Information [file 41467_2024_46592_MOESM3_ESM.pdf]

Inventory of Supporting Information

- **Supplementary Fig. 1.** Differential coexpression and upstream transcriptional regulators of the regulomes related to immune response and neuroinflammation
- **Supplementary Fig. 2.** Assessment of functional role of KDM1A in PMA/Ionomycin stimulated fetal astrocytes at 3h and 6h (n=3 biological replicates, n=2 technical replicates)
- **Supplementary Fig. 3.** Differential coexpression and upstream transcriptional regulators of the regulomes related to neuronal function.
- **Supplementary Fig. 4.** Expression profiles of five selected genes in all independent cohorts with qPCR (n = 6 per cohort, n=2 technical replicates) and RNAseq.
- **Supplementary Data 1:** Gene module summary
- **Supplementary Data 2:** Genes and modules
- **Supplementary Data 3:** Differential coexpression
- **Supplementary Data 4:** Functional annotation.
- **Supplementary Data 5:** Summary cohort comparison.
- **Supplementary Data 6:** Sample metadata information, Sequencing samples and functional studies samples
- **Supplementary Data 7:** Full list of genes included in the target gene panel
- **Supplementary Data 8:** Differential expression results for KCC2 and NKCC1 across the different cohort.
- **Supplementary Data 9:** Differential expression results on genes belonging to “GABA signalling pathway” (GO:0007214) across the different comparison (mTLE, FCD2b, TSC, and MTOR).
- **Supplementary Data 10:** Differential expression results on genes belonging to “glutamate receptor signalling pathway” (GO:0007215) across the different comparison (mTLE, FCD2b, TSC, and MTOR).
- **Supplementary Data 11:** Primers and sequence used for RT-PCR.
